# A Research Partnership to Enhance Postgraduate Pharmacy Residency Training Outcomes

**DOI:** 10.3390/pharmacy8030134

**Published:** 2020-07-31

**Authors:** Jennifer M. Bingham, Armando Silva Almodovar, Ann M. Taylor, David R. Axon, Milap C. Nahata, Sandra Leal, Terri Warholak, Nicole Scovis

**Affiliations:** 1 Tabula Rasa HealthCare Group, Tucson, AZ 85701, USA; SLeal@trhc.com (S.L.); NScovis@trhc.com (N.S.); 2 Institute of Therapeutic Innovations and Outcomes, College of Pharmacy, Ohio State University, Columbus, OH 43210, USA; Silvaalmodovar.1@osu.edu (A.S.A.); Nahata.1@osu.edu (M.C.N.); 3 College of Pharmacy, University of Arizona, Tucson, AZ 85721, USA; Taylor@pharmacy.arizona.edu (A.M.T.); Axon@pharmacy.arizona.edu (D.R.A.); Warholak@pharmacy.arizona.edu (T.W.)

**Keywords:** residency, research, post-graduate year-2, ambulatory care

## Abstract

Pharmacy residents must complete research as part of their program; however, challenges exist in providing experiences that result in successful research dissemination outcomes. A university-based research team, integrated into an ambulatory care pharmacy residency program aimed to improve presentation and publication rates of pharmacy resident research projects. Data on the number of postgraduate year-2 (PGY2) residents and their productivity were collected and summarized to assess progress. A total of 13 residents completed their residency over seven years. Each resident produced one regional presentation, and one national presentation beginning in year four. To date, three peer-reviewed papers have been published, with another one in-press. Responses from residents found lack of guidance, lack of data availability for projects and feedback fatigue were barriers to a positive research experience. To address these problems, a university-based research team was integrated to provide research mentor guided support, ensure study feasibility, and provide structured feedback. This program evaluation highlighted the integration of a PGY2 ambulatory care pharmacy residency with a designated, interprofessional university-based research team. Future work is warranted to reduce research-related barriers and formally evaluate resident post-program knowledge, skills, and subsequent dissemination rates.

## 1. Introduction

During postgraduate pharmacy training, second year ambulatory care residents are required to develop and present, in writing, a final research project suitable for publication as part of their longitudinal professional program activity requirements, upon completion of a 12-month program. However, submission for publication during the residency year is not required by the American Society of Health-System Pharmacists (ASHP) accrediting organization to successfully complete a postgraduate year-2 (PGY2) ambulatory care residency program [1]. While research experience is beneficial for trainees, challenges still remain for the faculty and affiliated organizations to adequately allocate the necessary amount and level of research training to residents, while balancing the resident’s time and competing priorities within a multifaceted PGY2 residency program. Whereas it differs among residency sites, most programs primarily focus on the development of clinical skills, rather than emphasizing in-depth research expertise that would be more typical of a graduate research or fellowship program. Hoffman et al. reported that one of the most challenging tasks for residency directors was the implementation of a successful research program, due to challenges with regulatory requirements [2]. Areas for improvement included an organizations’ preparedness to implement research experiences, residents’ low interest in research, program structure-related issues, effective mentorship [3], and low publication rates [4]. Thus, more work is needed to address gaps in program implementation, to improve postgraduate resident experiences and outcomes.

Presently, there are notable challenges for program staff and trainees alike [3]. Some reported barriers include: programs inadequately prepared to offer formal training in conducting research or having insufficient qualified research mentors [5,6,7]; failing to develop a realistic one-year (residency) timeline; not obtaining institutional review board (IRB) approval; difficulty progressing through the publication process; difficulty with data collection; and, inadequately expanding trainee professional opportunities [3,8]. Residency program directors reported that roughly 64% of postgraduate year-1 (PGY1) and PGY2 pharmacy residents failed to publish their research projects within 12 months post-residency [3]. Finally, residency program directors and residents cited two main publication barriers, including study design quality and time constraints [3]. Shafeeq et al. also found that residents’ perceived publication success was related to: a higher degree of self-motivation to publish; programs providing adequate manuscript preparation training; as well as allowing adequate time for manuscript completion; and post-residency mentoring support [4]. 

From a programmatic standpoint, the goal of integrating a designated, university-based research team was to expand trainees’ knowledge, broaden their research experiences, meet the American Society for Health-System Pharmacists (ASHP) requirements for residency programs [1], and improve presentation and publication rates of pharmacy resident research projects. According to Irwin et al. [3], program directors and trainees reported that research projects offered opportunities for important skill development (management, administrative, time management) and better preparation for conducting future research [3]. Moreover, Shafeeq et al. noted that research activities served to strengthen residents’ written and verbal communication as well as problem-solving skills [4]. Kari et al. found that clinical pharmacy research teams were successful at improving publication of pharmacy resident research projects in peer-reviewed journals [9]. Despite these noted accomplishments, barriers still remain.

The purpose of this paper was to describe the development, evolution, and outcomes of a PGY2 ambulatory care pharmacy residency program research learning experience, in collaboration with a designated, university-based research team aimed to improve presentation and publication rates of pharmacy resident research projects. This paper also highlights areas of success and those requiring improvement for the community of existing or upcoming pharmacy residency programs. 

## 2. Materials and Methods

### 2.1. Data Collection and Analysis

This program evaluation used previously collected quality improvement outcomes data from June 2012 to June 2019 provided by the residency program director. Written summative evaluation data from both the residents and preceptors were systematically collected at the end of the residency program year by the residency program director through a web-based residency evaluation system. The variables of interest collected by the residency program director included: (1) number of presentations; (2) number of publications; (3) program feedback provided by the resident to the preceptor; (4) program feedback provided by the preceptor to the resident; (5) proposed changes for the next residency program year; and (6) number of residents each program year. This program evaluation was exempt from institutional review board review, as it was deemed a quality improvement project. Descriptive statistics were used to present the summative evaluation data. 

### 2.2. Description of the Postgraduate Year Two (PGY2) Ambulatory Care Pharmacy Program Residency Program

The PGY2 ambulatory care pharmacy residency program in southern Arizona was established in 2012 and was fully accredited by ASHP in 2019, for an additional 8 years. The program was designed to prepare the resident to be a successful ambulatory care pharmacist upon completion of the program. Each year, the program supervised 1 to 2 PGY2 ambulatory care pharmacy residents. This team consisted of 1 residency program director, 1 orientation preceptor, 1 professional activities preceptor, 3–4 chronic disease management preceptors, 2 primary care preceptors, 1 geriatric pharmacy preceptor, 2 business and administration preceptors, 2 elective preceptors, and 2 research preceptors. 

### 2.3. Description of the Postgraduate Year Two (PGY2) Residency Research Team

The PGY2 ambulatory care pharmacy residency program research learning experience employed innovative approaches to foster successful knowledge and skill acquisition, and encompassed multiple components to facilitate and promote valuable, research-related, postgraduate and lifelong learning experiences. Each resident completed a 12-month longitudinal research experience, and was provided one-half day each week dedicated to complete research related activities and avoid time constraints. The research learning experience team consisted of: a PGY2 ambulatory care pharmacy resident; a residency program director (JB, NS); research experience preceptors (JB, NS); a university-based research director (TW); a medical editor (AT); and graduate research student mentor(s). The residency program director provided oversight and guidance throughout the resident’s year-long learning experience. The preceptor and residency program director’s role, based on the ASHP resident feedback method, integrated: (1) direct instruction; (2) modeling; (3) coaching; and (4) facilitating [10]. 

### 2.4. Description of the University-Based Research Team

**Figure 1:**
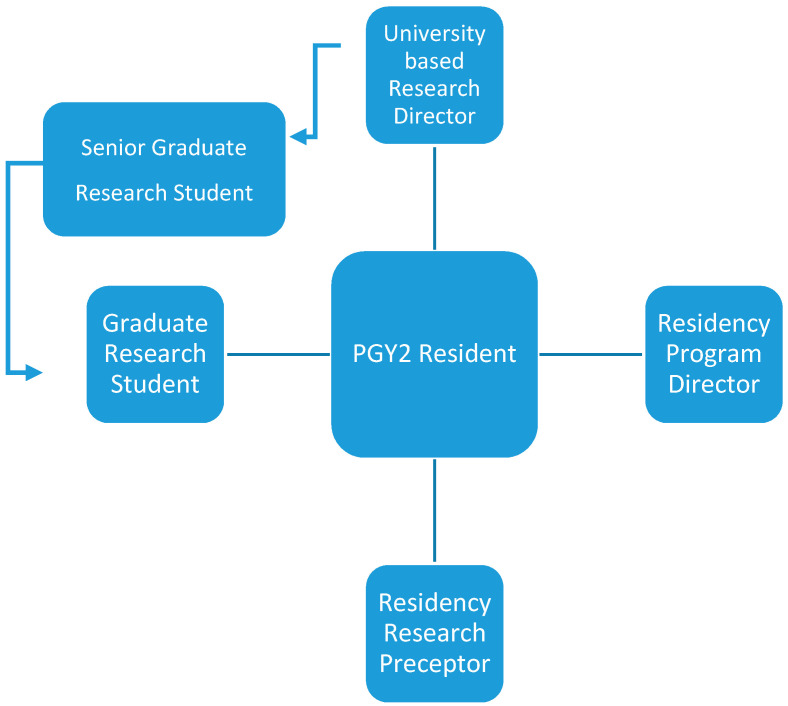
Description of Layered Mentoring within Research Team.

The university-based research director (TW), medical editor (AT), and graduate research student mentor(s) provided guidance and mentorship in research design, data collection and analysis, evaluation, and dissemination. The university-based research director was a tenured faculty member at a southern Arizona accredited college of pharmacy, with expertise in health and pharmacoeconomic outcome research. Each year, the university-based research director was required to supervise 3 to 5 doctoral students. This team typically published between 5 and 12 peer-reviewed papers per year in high-impact journals, in addition to delivering several poster and podium presentations. The senior graduate research students served as the graduate research student mentor to the PGY2 resident. Through this layered mentoring approach (Figure 1), the graduate research student mentor helped the resident to complete each task in the research project. It is important to note, the resident solely interacted with their paired graduate research student mentor, to avoid feedback fatigue from the entire university-based research team. 

### 2.5. Description of the Research Learning Experience 

Residents were expected to demonstrate ability to conduct a research project by successfully achieving the objectives listed in Table 1. The preceptor evaluated the resident on a quarterly basis throughout the residency year, using the summative evaluation scale provided by ASHP to identify areas for improvement in their learning experience [10]. Preceptor feedback was solicited at quarterly residency advisory committee meetings, and through quarterly assigned summative evaluations. The preceptor scored the resident’s performance on each objective in the summative evaluation as either: (1) not achieved; (2) needs improvement; (3) satisfactory progress; (4) achieved; or, (5) achieved for the residency. If the resident did not progress at the required rate to fully achieve the objective, additional verbal and/or written feedback was provided by the preceptor to the resident [10]. 

Residents were immersed in this team-based research learning experience within four weeks of beginning their residency program, with the goal of presenting their completed project at both a regional and a national conference and publishing it in a peer-reviewed journal. The PGY2 pharmacy residency program research learning experience was structured to guide residents through the process in a logical sequence, and to facilitate completion of goals in a timely manner. 

During Month 01, the resident collaborated with the university-based research team to select a project, aligning with the organization’s mission, vision, and strategic growth initiatives. The resident also attended an onsite Institutional Review Board (IRB) orientation session at the Human Subjects Protection Program office and completed all relevant collaborative institutional training initiative (CITI) courses [11,12]. To improve pharmacy residents’ confidence, competence in research idea conception and attitudes towards research, they were introduced to the “A Structured Program to Guide Residents’ Experience in Research” (ASPIRE) program developed by Kaiser Permanente Colorado in the year 2019 [13]. Residents completed the first ASPIRE module focusing on: (1) organizing background literature; (2) drafting study design; (3) selecting inclusion and exclusion criteria; and, (4) creating a data needs worksheet [13].

During Month 02, the resident drafted a proposal for peer-review and approval by their respective research team. Given the short timeframe to prepare and submit a conference abstract, the resident simultaneously began drafting a conference abstract for both a national and regional meeting. The resident also completed the second and third ASPIRE modules, emphasizing study design, population, procedures, data elements (e.g., sample size calculations), and data analysis (with a special emphasis on biostatistics). 

In Month 03, the proposal content was used to draft either the Determination of Human Research Form or the Application for Human Research form [12], depending on the project, which was then submitted to the university IRB for approval. Once the resident received the IRB approval letter of correspondence, the conference abstract was prepared for submission in the “Research in Progress” category for final peer-review and approval by the medical editor one month prior to the submission deadline. 

In Month 04, once all aforementioned approvals were granted, the resident completed the online conference abstract submission. The resident further refined the statistical analysis approach with the graduate research student mentor, and the research preceptor ensured that all categories of the data dictionary were readily available for the collection phase. 

In Months 05 and 06, the resident began data collection and analysis, leading the team in identifying relevant outcomes (results) and discussion points. Conversations among the resident, research preceptor and graduate student research mentor(s) then ensued, regarding manuscript content, tables and figures, and potential journals to consider for submission.

During Months 07 and 08, once the resident’s presentation abstract was accepted, the resident was expected to draft poster/slide content, using a standardized template developed to guide poster/slide content and graphic creation. The content was collectively peer-reviewed within the research team, and once approved by the medical editor, was transposed to a poster/slide template, for printing prior to the conference. 

During the later Months (09–12) of the PGY2 pharmacy residency program research learning experience, the resident presented the research poster/slides at the selected conference(s); and audience feedback regarding the poster/slides was incorporated into the manuscript discussion and limitation sections. During this time, the research team peer-reviewed and provided mentorship as the resident finalized the manuscript sections. The manuscript then underwent final medical editor approval. The goal was to submit the manuscript to a peer-reviewed journal by completion of the residency program. Finally, the residency program director and resident met to discuss their research learning experience, where the resident had an opportunity to provide verbal feedback on their experiences. The residency program director completed qualitative documentation of verbal feedback to record resident progress and provide direction to the residents and vital information for the residency program director and future preceptors. Documentation was a required element of pharmacy residency standards [10]. This information was then shared with the research team, and quality improvement changes for the following year were planned for implementation. 

The residency advisory committee met every quarter to discuss continuous quality improvement initiatives to improve the learning experience of the resident, develop and implement program improvement activities in response to the results of the resident assessment of the research learning experience, and evaluate whether residents were successfully fulfilling the purpose of a PGY2 pharmacy residency program through graduate tracking. The committee consisted of core preceptors, residents, and the residency program director. Each preceptor offered suggestions regarding ways to improve the resident research learning experience the following year. Upon completion of the research learning experience, the research preceptors reflected on the summative feedback provided by the resident, to further implement solutions for these areas of improvement. 

## 3. Results

A total of 13 residents completed the PGY2 ambulatory care pharmacy residency program research learning experience over seven years (two residents per year, with only one resident in year six). All residents that started the program successfully achieved the objectives for the residency research learning experience listed in Table 1, and completed the 12-month program. Each resident produced one regional presentation in years one through three; beginning in year four, they also delivered one national presentation. To date, three peer-reviewed papers have been published [14,15,16]; and, one is awaiting galley proofs from a peer-reviewed journal. Early feedback from residents included a lack of guidance, which was subsequently addressed by integrating an academic-based research team to provide mentorship. Later feedback included a lack of data availability for chosen projects and overwhelming volumes of feedback, which were addressed by introducing a pre-approval process to ensure study feasibility and providing structured feedback over time. A summary of the results by year of the program is provided (Table 2).

Each year, the pharmacy residency research projects ranged in study design and complexity appropriate to the options available and interests of the resident. Studies employed quantitative and qualitative techniques and included surveys, focus groups, database analyses, systematic reviews, and program evaluations, among others, using retrospective, cross-sectional, and prospective perspectives.

## 4. Discussion

This program evaluation highlights the integration of designated, collaborative research team approaches employed in a university-based PGY2 ambulatory care pharmacy residency program, designed to improve presentation and publication rates of pharmacy residency research projects. 

The collaborative training processes outlined above addressed an important gap in the literature regarding the need for formalized research training for residents. Specifically, the academic institution and their affiliates provided formalized training to respective trainees, utilizing strategies to improve confidence, as well as competence [17]. Given the typically low post-completion knowledge and skills reported in the literature [6,18], it is feasible that providing more formalized training to postgraduate year-2 residents as presented here, and in a postgraduate year-1 residency program [19], may result in trainees’ greater research knowledge and skills post-program completion. Furthermore, Irwin et al. found that this knowledge gap was a principal barrier in trainee publication [3]. Thus, future work is warranted to evaluate trainees’ pre- and post-program research-related knowledge and skills. 

The formal training program experienced multiple successes. Based on former resident feedback, residents are now paired with graduate research student mentors early in the research experience, to: (a) conduct one-on-one focused sessions to help guide the research study design, data analysis and interpretation; (b) provide assistance and support to residents; and (c) mentor residents to help hone their knowledge and skills. Moreover, the layered mentoring process [20,21], bridged gaps and provided opportunities for respective residents to achieve desirable outcomes (e.g., project completion, publication) [3,17].

The university-based research team was able to mentor and guide residents in all research phases, especially study design and data analysis, to ensure use of appropriate statistical tests and interpretation of results. The effects of the pharmacy residency mentorship program approach parallel other studies and proved to be beneficial in this PGY2 ambulatory care residency training program [22]. However, processes from year 07 were identified as needing improvement, such as live edits on the initial abstract, which proved overwhelming for residents. This process was introduced to address previous feedback that individual comments from multiple members of the research team produced conflicting recommendations. Subsequently, abstract peer-review is now conducted in multiple, shorter sessions, and this process will be monitored and further refined as necessary, to optimize the experience. This was the first residency year (2019–2020) of integrating the self-paced ASPIRE modules, thus, no resident feedback is available. These modules may provide new information for some, while serving as a brief refresher course for others more familiar with research methods. These modules may improve residents’ overall research knowledge, skills and perceived ability to communicate respective research concepts in disseminating their results (e.g., poster/podium presentations, manuscripts). Yet, evaluating residents’ research-related competencies and outcomes (e.g., presentations, publications) within this PGY2 pharmacy residency program research learning experience is warranted. 

The various challenges, revisions, and successes of this PGY2 residency offer ‘lessons learned’ to upcoming and seasoned program directors. The results of this collaborative research team approach also lend evidence to the value of incorporating key aspects of health economic and outcomes research training into residency programs [23]. One novel aspect of this program was establishing the early partnership with a university-based research team who provided mentorship to residents. Over time, this mutually beneficial relationship resulted in the dissemination of important new health services and outcomes to benefit the research team and improve the quality of presentations and publications. The research learning experience evolved, to ensure that the resident’s valuable work proceeded to publication, as the resident often had competing priorities upon completion of the PGY2 program, and inadequate time to prepare their former residency project for publication. Given this tight timeframe, residents successfully partnered with the university-based research team, to facilitate the research process and provide mentorship and expertise. Secondly, this highlights the importance of a well-structured program timeline, to ensure tasks are completed in a logical order and deadlines (e.g., conference abstract and manuscript submissions) are met. Feedback from the review process emphasized the need for frequent peer-review, to ensure the program continues to meet the residents’ needs. Our results also support the importance of research during pharmacy residency training [24]. Finally, the ‘lessons learned’ from the PGY2 research learning experience was adapted for use in a two-year postgraduate pharmacy research fellowship with a partner institution. The fellow also had access to a graduate research student mentor and received layered mentoring from the same university research team. Several components of the PGY2 ambulatory care pharmacy residency program research learning experience proved valuable in the fellowship program, resulting in four national presentations and five peer-reviewed papers during the two-year fellowship [25,26,27,28,29]. 

### Limitations

Despite the versatility of the partnership, this program evaluation only describes a small sample of residents. Thus, these findings are not generalizable to all PGY2 ambulatory care pharmacy residency programs or research fellowships. In addition, this review was not able to capture evidence to describe whether other confounding factors, specifically one’s personal publication goals, influenced the ability to successfully disseminate research findings.

## 5. Conclusions

This program evaluation highlights the integration of a collaborative research team approach between a PGY2 pharmacy residency program and a university-based research team, and lends preliminary evidence to support its value in improving presentation and publication rates of pharmacy residency research projects. The collaborative training processes addressed an important gap regarding the need for formalized research training for PGY2 residents, and may provide useful insight for residency directors facing challenges with the research component of their programs, or to programs with more than one resident in need of structured research mentorship. Yet, a more formalized assessment of residency graduates is warranted to investigate their skill acquisition and subsequent presentation and publication rates beyond the completion of residency training.

## Figures and Tables

**Table 1:** Postgraduate Year Two (PGY2) Pharmacy Program Research Experience Required Objectives [10].

Objective	Description of Activities
Analyzing	Identify a scholarly research question related to clinical practice, education, or healthcare to be completed within the PGY2 ambulatory care pharmacy residency year. Implement the research project.
Creating	Develop a research protocol for the specified project. Effectively develop and present a final project report acceptable for publication.
Evaluating	Collect and evaluate data for the research project. Assess for need to make changes based on research project.

**Table 2:** Postgraduate year-2 pharmacy (PGY2) residency program research learning experience productivity, program feedback, and changes made during quality improvement cycles.

Program Year	Research Dissemination	Program Feedback	Proposed Changes for the Next Program Year
Year 1 2012–2013	2 regional podium presentations (RPPs)	Residents felt they lacked sufficient direction/mentorship Preceptors did not feel they had sufficient expertise to mentor residents, and were offered preceptor development opportunities Preceptors felt the resulting resident projects were of low quality	Preceptors make initial plans to enhance research learning experience with mentorship opportunities
Year 2 2013–2014	2 RPPs 1 publication	Residents felt the research elective helped provide guidance Residents developed their own project ideas, and preceptors felt this led to a poor correlation between research projects and the organizations’ needs Preceptors felt there was still inconsistent study quality	Residents to collaborate with university-based research team to increase research mentoring and study quality
Year 3 2014–2015	2 RPPs 1 publication	Research team members and residents felt the roles of resident, graduate student, resident preceptor, graduate student advisor and others needed further clarification to facilitate better collaboration Preceptors were concerned that residents were lacking exposure to different specialty areas given their presentations were limited to regional conferences only Preceptors felt there was better study quality	Delineate team member roles Formalize 1:1 pairing of resident to graduate student Add an extra presentation at a national pharmacy conference to resident expectations
Year 4 2015–2016	2 RPPs 2 NPPs	Residents and preceptors felt that the collaboration with the research team provided a real-world collaboration experience Residents felt it was difficult to manage research	Clarify that the resident was the lead person for their residency project team
Year 5 2016–2017	2 RPPs 2 NPPs	Residents felt the complicated, multiple step research proposal peer-review process resulted in conflicting feedback which they did not know how to address	Plan live proposal peer-review and abstract editing sessions to gain consensus on feedback
Year 6 2017–2018 *	1 RPP 1 NPP 1 publication	Residents felt the lack of available data sets upon selecting their project resulted in analysis delays.	Pre-approve project ideas to ensure data availability
Year 7 2018–2019	2 RPPs 2 NPPs 1 article in -press 1 article under review	Residents and preceptors felt that live editing of abstracts resulted in an overwhelming amount of feedback Research team members felt that residents lacked a basic understanding of research methods	Change abstract peer-review meetings to a stepwise approach to prevent overwhelming amounts of feedback Add the ASPIRE modules to teach the residents baseline methodology

* N = 2 residents/year except for year 6 where N = 1RPP = Regional podium presentation; NPP = National poster presentation; ASPIRE = A Structured Program to Guide Residents’ Experience in Research [13].
